# FGFR2 and favorable survival outcomes in resected poorly cohesive cell gastric cancer: Analysis from FGFR2 protein overexpression and genetic variation

**DOI:** 10.1371/journal.pone.0349408

**Published:** 2026-06-01

**Authors:** Yun Ji Lee, Inuk Jung, Jin Ho Baek, Jong Gwang Kim, Ki Bum Park, Ji Yeon Park, Oh Kyoung Kwon, An Na Seo, Moonsik Kim, Byung Woog Kang

**Affiliations:** 1 Department of Hematology/Oncology, Yeungnam University College of Medicine‌‌, Daegu, Republic of Korea; 2 School of Computer Science and Engineering, Kyungpook National University, Buk-gu, Daegu, Republic of Korea; 3 Department of Oncology/Hematology, Kyungpook National University Chilgok Hospital, School of Medicine, Kyungpook National University, Kyungpook National University Cancer Research Institute, Daegu, Republic of Korea; 4 Department of Surgery, Kyungpook National University Chilgok Hospital, School of Medicine, Kyungpook National University, Daegu, Republic of Korea; 5 Department of Pathology, Kyungpook National University Medical Center, Kyungpook National University School of Medicine, Daegu, Republic of Korea; University of Vermont, UNITED STATES OF AMERICA

## Abstract

**Purpose:**

Poorly cohesive cell gastric cancer has aggressive and heterogeneous characteristics. This study investigated the clinical significance of fibroblast growth factor receptor 2 expression and genetic variations in patients with PCC-GC.

**Materials and Methods:**

We retrospectively collected 209 surgically resected stage II and III PCC-GC cases. After FGFR2 immunostaining, we analyzed clinical data and performed targeted sequencing to assess their impact on patient survival.

**Results:**

Among 209 patients, 89 (42.6%) were classified as stage II and 120 (57.4%) as stage III. FGFR2 overexpression varied by stage, with FGFR2 positivity observed in 61.5% of stage II cases, while FGFR2-negative cases were predominant in stage III patients (60.1%) (P = 0.037). Moreover, lymph node involvement was associated with FGFR2 expression (P = 0.009). FGFR2 positivity significantly correlated with improved disease-free survival (DFS) and overall survival and remained an independent favorable prognostic factor for DFS in multivariate analysis (P = 0.022). Targeted next-generation sequencing was performed in five selected cases, *FGFR2* amplification or pathogenic alterations were not found.

**Conclusion:**

This study shows that FGFR2 expression was independently associated with improved DFS in PCC-GC. These findings suggest that FGFR2 may serve as a prognostic biomarker in patients with PCC-GC.

## Introduction

As the molecular complexity of gastric cancer (GC) becomes better understood, increasing attention has focused on its histological subtypes, which exhibit distinct biological and clinical characteristics [1]. Among them, poorly cohesive GC (PCC-GC) is recognized as a distinct subtype, marked by diffuse growth, pronounced molecular heterogeneity, and intrinsic resistance to standard therapies [1,2]. These pathological features contribute to poor clinical outcomes, making PCC-GC one of the most aggressive GC subtypes [3]. Although several molecular alterations have been identified in PCC-GC, their prognostic value remains unclear [4]. Moreover, subgroup analyses have shown limited responses to standard therapies, highlighting the urgent need for novel molecular targets and predictive biomarkers to improve therapeutic outcomes in this subset of patients [3].

Fibroblast growth factor receptor 2 (FGFR2) is a receptor tyrosine kinase involved in tumor proliferation, angiogenesis, and metastasis [5]. Aberrant FGFR2 signaling, including gene amplification, activating mutations, and FGFR2 overexpression, is associated with poor prognosis in several malignancies, and FGFR2-targeted therapies such as bemarituzumab have shown promise in clinical trials for FGFR2-overexpressing GC [5–7]. However, the role of FGFR2 expression in PCC-GC remains largely unexplored [5,8]. Given the heterogeneity of PCC-GC and its resistance to conventional treatments, understanding the clinical significance of FGFR2 expression may aid in identifying potential therapeutic strategies [6].

Therefore, this study aimed to investigate the relationship between FGFR2 expression, genetic variation, and prognosis in PCC-GC, contributing to efforts to develop improved biomarker-based therapeutic strategies for PCC-GC.

## Materials and methods

### Study population‌‌

We retrospectively analyzed data from 230 patients with stage II and III PCC-GC who underwent surgical resection at Kyungpook National Chilgok Hospital from April 2011 to May 2022. Among these, 209 patients who received postoperative adjuvant chemotherapy were included in the final analysis. Tumors were staged according to the 8th edition of the American Joint Committee on Cancer (AJCC) system [9]. Clinical and pathological data such as age, sex, tumor location, lymph node status, tumor stage, type of surgical procedure, and serum levels of carcinoembryonic antigen and carbohydrate antigen 19−9 were extracted from the hospital’s electronic medical records. Postoperative adjuvant chemotherapy, consisting of either tegafur/gimeracil/-oteracil (S-1) or capecitabine/oxaliplatin, was initiated 4–6 weeks after surgery in patients with stage II or III. The study was approved by the Institutional Review Board of Kyungpook National University Medical Center (IRB No. KNUMC 2022-11-004).

### Pathologic evaluation and tissue microarray construction

Tumor specimens were fixed in 10% neutral-buffered formalin, embedded in paraffin, and sectioned into 4 μm slices. Hematoxylin and eosin staining were performed, and slides were reviewed by a gastrointestinal pathologist (MSK), who selected representative tumor areas for tissue microarray (TMA) construction. Following histological assessment, two 2-mm cores were extracted from formalin-fixed, paraffin-embedded (FFPE) tissue blocks and used to construct TMAs for immunohistochemical analysis.

### Immunohistochemistry and interpretation

**Fig 1:**
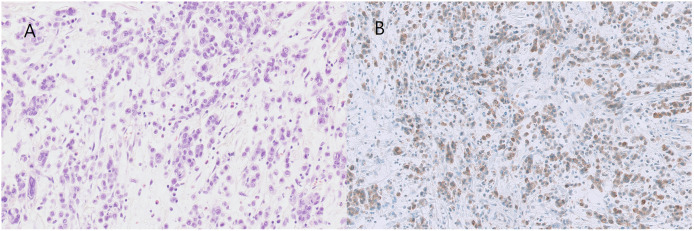
Representative photos of FGFR2-overexpressing poorly cohesive carcinoma (PCC-GC). **(A)** Hematoxylin and eosin (H&E) staining, **(B)** Immunohistochemical staining for FGFR2. Original magnifications: (A) and **(B)**: × 200.

TMA FFPE slices were deparaffinized and rehydrated with xylene and alcohol, then incubated with antibodies against FGFR2 (rabbit monoclonal, clone SP273, dilution 1:100, Abcam, Cambridge, UK). The sections were chromogenically visualized using an ultraView Universal DAB Detection Kit (Ventana Medical Systems) or EnVision FLEX/HRP (Agilent Technologies) and counterstained with hematoxylin. FGFR2 overexpression was defined as ≥5% of tumor cells showing moderate (2+) to strong (3+) membranous or cytoplasmic staining (Fig 1) [7]. Immunohistochemical staining was independently evaluated by two experienced pathologists, and discrepancies were resolved by consensus.

### Targeted sequencing

*FGFR2* mutational and amplification status was assessed using targeted next-generation sequencing (NGS). Five representative cases were selected for sequencing analysis using both fresh frozen and FFPE samples. Genomic DNA was extracted, and library preparation was performed using the CancerSCAN Library Preparation Kit and CancerSCAN IO+ panel (GENINUS Inc., Seoul, Republic of Korea), targeting 407 cancer-associated genes. Sequencing was performed on the Illumina NextSeq 550Dx platform (Illumina, Inc., California, USA). The NGS samples were aligned to the human reference genome hg19 (GRCh37.p13). Mutations were called using the standard MuTect pipeline using the following databases: dbSNP, Korean Variant Archive (KOVA), Korean Reference Genome Database (KRGDB, v622, v1100), COSMIC (v66) and ClinVar. DbSNP, KOVA, KRGDB were used to remove variants that are likely to be germline. COSMIC, ClinVar databases were used to annotate biological and clinically related variants.

### Statistical analysis

Associations between clinicopathologic parameters were assessed using the chi-square test. There were no significant missing data for the variables included in the analysis. Disease-free survival (DFS) was defined as the time from surgery to recurrence or death from any cause, and overall survival (OS) as the time from surgery to death from any cause. Survival outcomes were estimated using Kaplan–Meier methods, and differences between groups were compared using the log-rank test in univariate analysis. Statistically significant variables in univariate analysis were further evaluated using the Cox proportional hazards model to identify independent prognostic factors in multivariate analysis. Multivariable Cox proportional hazards models were used to adjust for potential confounders, including age, sex, and tumor stage. A p-value <0.05 was considered significant. All analyses were performed using SPSS software, version 29.0 (IBM, Armonk, NY, USA).

### Ethics approval and consent to participate

The Institutional Review Board of Kyungpook National University Medical Center (IRB No. KNUMC 2022‑11‑004) approved this study. Following this approval in November 2022, the data were accessed for research purposes between December 2022 and January 2023. All data were anonymized prior to analysis, and the authors did not have access to information that could identify individual participants during or after data collection. All procedures in this study that involved human participants were performed in accordance with the ethical standards of the institutional and/or national research committee and with the 1964 Declaration of Helsinki and its later amendments or comparable ethical standards. Written informed consent was waived by the Institutional Review Board due to the retrospective nature of this study.

## Results

### Patients and characteristics

The clinical characteristics of the 209 patients are summarized in Table 1. The median age at diagnosis was 63 years (range: 30–84 years), with 112 (53.6%) male. The most common tumor location was the stomach body (134 patients, 64.1%), followed by the antrum (59 patients, 28.2%). In total, 104 (49.8%) patients underwent total gastrectomy, while 98 (46.7%) underwent distal gastrectomy. After surgery, 89 patients (42.6%) were classified as stage II and 120 (57.4%) as stage III. Histologically, 99 (47.4%) patients had PCC-SRC, 71 (34.0%) had PCC-NOS, and 39 (18.7%) had PCC-Mixed.

**Table 1:** Baseline characteristics of patient cohort.

Factors	N = 209
Age, years	
Median (range)	63(30.0-84.0)
Gender	
Male	112(53.6)
Female	97 (46.4)
Tumor Location	
Cardia	8(3.8)
Fundus	8(3.8)
Body	134(64.1)
Antrum	59(28.2)
Pylorus	0(0)
pTNM stage	
II	89(42.6)
III	120(57.4)
T stage	
T1	14(6.7)
T2	17(8.1)
T3	55(26.3)
T4	123(58.9)
N stage	
N0	53(25.4)
N1	32(15.3)
N2	45(21.5)
N3	79(37.8)
Surgery	
Total gastrectomy	104(49.8)
Distal gastrectomy	98(46.7)
Other	7(3.3)
Pre-Op CEA	
<5	192(91.9)
≥5	17(8.1)
Pre-Op CA 19−9	
<37	183(87.6)
≥37	26(12.4)
Pathology	
PCC-SRC	99(47.4)
PCC-NOS	71(34.0)
PCC-Mixed	39(18.7)
Recurrence	76(36.4)

TNM, tumor-node-metastasis; Op, operation; CEA, *carcinoembryonic antigen; CA 19−9, carbohydrate antigen 19−9;* NOS, not otherwise specified; PCC, poorly cohesive carcinoma; SRC, signet ring cell carcinoma.

### Relationship between FGFR2 expression and clinicopathologic features

The association between FGFR2 expression and clinicopathologic features is summarized in Table 2. Among the 209 patients, 26 (12.4%) were FGFR2-positive, while 183 (87.6%) were FGFR2-negative. FGFR2 overexpression varied by stage: 61.5% of stage II cases were FGFR2-positive, while 60.1% of stage III cases were FGFR2-negative (P = 0.037). Moreover, lymph node involvement correlated with FGFR2 expression (P = 0.009), with FGFR2 positivity more frequent in patients without nodal metastasis (N0, 42.3%) than in those with advanced nodal stage (N3, 11.5%).

**Table 2:** The association between FGFR2 expression and clinicopathologic features.

	FGFR2		
Variables	Negative	Positive	
	N = 183	N = 26	p-value
Age, years			
<65	136(74.3)	18(69.2)	0.582
≥65	47(25.7)	8(30.8)	
Gender			
Male	96(52.5)	16(61.5)	0.385
Female	87(47.5)	10(38.5)	
Tumor Location			
Cardia	7(3.8)	1(3.8)	0.181
Fundus	7(3.8)	1(3.8)	
Body	122(66.7)	12(46.2)	
Antrum	47(25.7)	12(46.2)	
Pylorus	0(0.0)	0(0.0)	
pTNM stage			
II	73(39.9)	16(61.5)	0.037
III	110(60.1)	10(38.5)	
T stage			
T1	13(7.7)	1(3.8)	0.851
T2	14(7.7)	3(11.5)	
T3	48(26.2)	7(26.9)	
T4	108(59.0)	15(57.7)	
N stage			
N0	42(23.0)	11(42.3)	0.009
N1	25(13.7)	7(26.9)	
N2	40(21.9)	5(19.2)	
N3	76(41.5)	3(11.5)	
Lauren classification			
Interstitial	2(1.1)	0(0)	0.438
Diffuse	172(94.0)	26(100.0)	
Mixed	9(4.9)	0(0)	
Surgery			
TG	93(50.8)	11(42.3)	0.356
DG	83(45.4)	15(57.7)	
Other	7(3.8)	0(0)	
Pre-Op CEA			
<5	166(90.7)	26(100.0)	0.105
≥5	17(9.3)	0(0)	
Pre-Op CA 19−9			
<37	159(86.9)	24(92.3)	0.433
≥37	24(13.1)	2(7.7)	
Pathology			
PCC-SRC	89(48.6)	10(38.5)	0.169
PCC-NOS	58(31.7)	13(50.0)	
PCC-Mixed	36(19.7)	3(11.5)	

FGFR, fibroblast growth factor receptor; TNM, tumor-node-metastasis; TG, total gastrectomy; DG, distal gastrectomy; Op, operation; CEA, *carcinoembryonic antigen; CA 19−9, carbohydrate antigen 19−9;* NOS, not otherwise specified; PCC, poorly cohesive carcinoma; SRC, signet ring cell carcinoma.

### Effect of FGFR2 expression on survival outcomes

**Fig 2:**
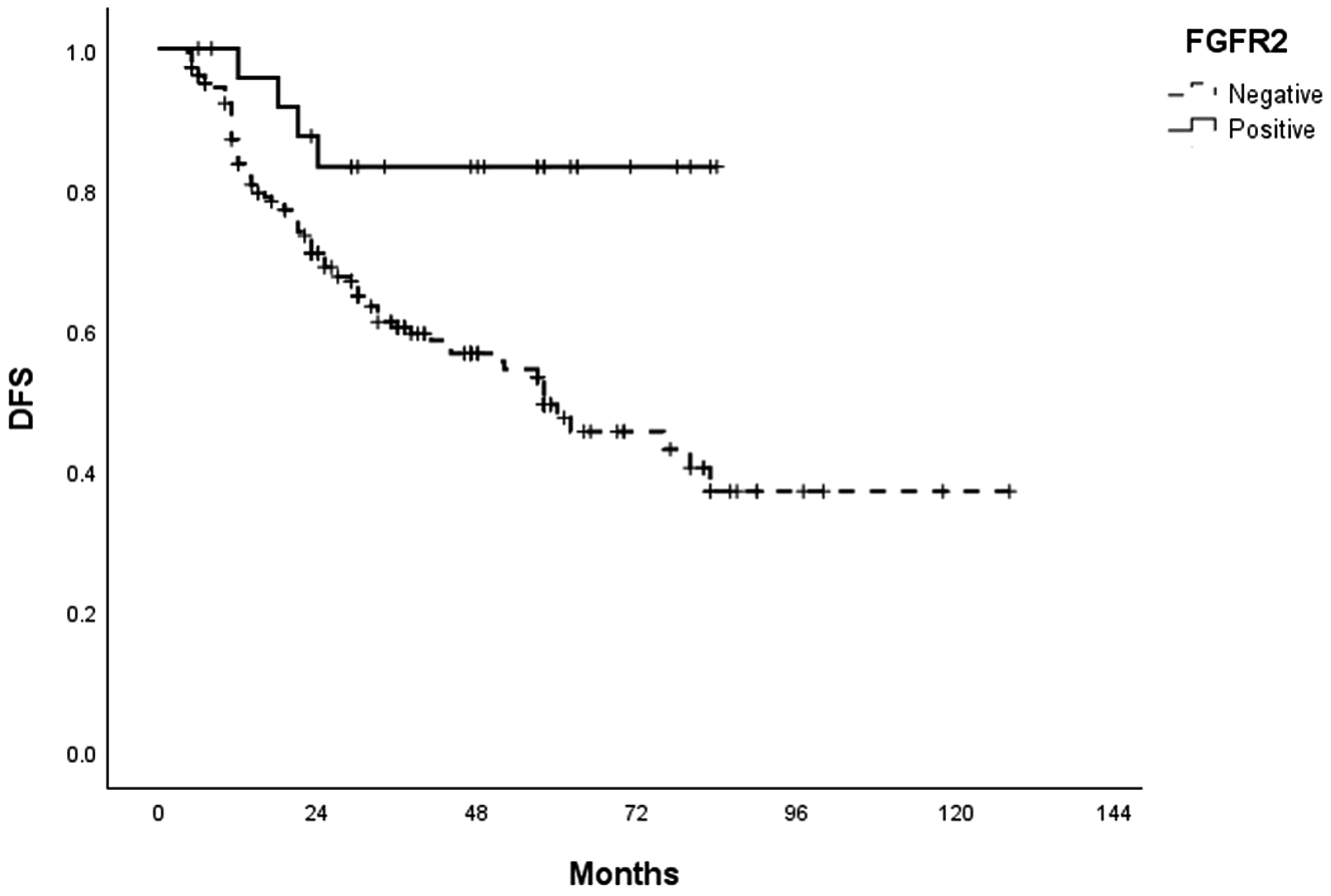
Kaplan-Meier survival curves for disease-free survival (DFS) according to the expression of FGFR2 in patients with PCC-GC. p-value = 0.007 (Log Rank).

**Fig 3:**
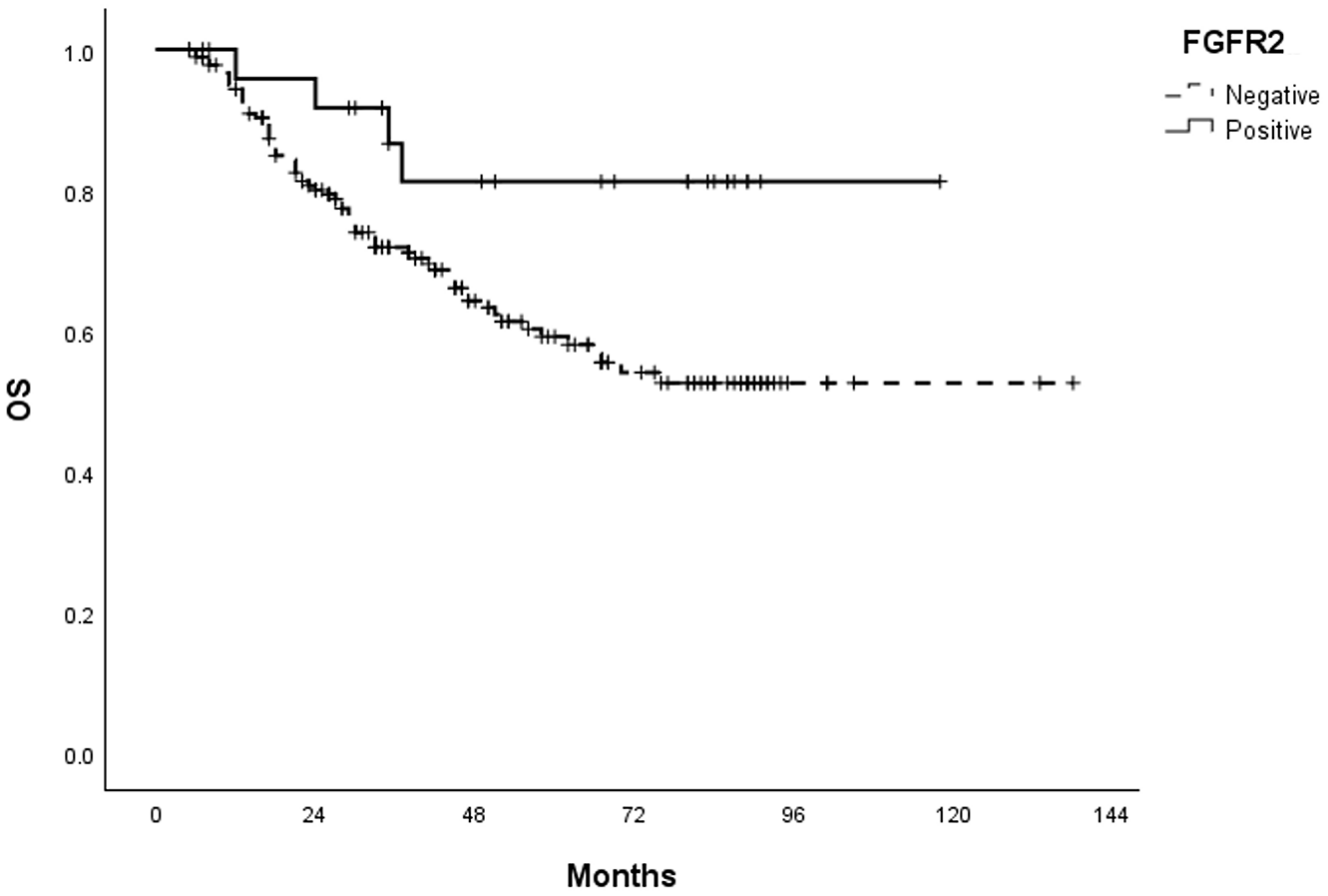
Kaplan-Meier survival curves for overall survival (OS) according to the expression of FGFR2 in patients with PCC-GC. p-value = 0.030 (Log Rank).

The median follow-up duration for survival analyses was 38.9 months. During the study period, 76 patients (36.4%) experienced recurrence. Notably, in the univariate analysis, FGFR2 positivity significantly correlated with favorable DFS (HR = 0.279, 95% CI = 0.102–0.763, P = 0.013) (Table 3). Kaplan–Meier survival curves revealed that FGFR2-positive tumors were associated with improved DFS compared to FGFR2-negative tumors (P = 0.007) (Fig 2). FGFR2 overexpression also significantly impacted OS, favoring superior outcomes (P = 0.030) (Fig 3).

**Table 3:** Univariate and multivariate analyses of disease-free survival (DFS).

Variables	Category	Disease free survival					
		Univariate analysis			Multivariate analysis		
		HR	95% CI	p-value	HR	95% CI	p-value
Age	<65 vs. ≥ 65	0.400	0.257-0.622	<0.001	0.380	0.244-0.591	<0.001
Stage	II vs. III	0.281	0.165-0.479	<0.001	0.304	0.177-0.522	<0.001
Sex	Male vs. Female	1.519	0.972-2.373	0.067	1.420	0.903-2.234	0.129
FGFR2	Positive. vs. Negative	0.279	0.102-0.763	0.013	0.306	0.111-0.840	0.022

HR, hazard ratio; CI, confidence interval, FGFR, Fibroblast growth factor receptor.

Multivariate analysis using the Cox proportional hazards model confirmed FGFR2 expression as an independent predictor of longer DFS (HR = 0.306, 95% CI = 0.111–0.840, P = 0.022). Age (P < 0.001) and stage (P < 0.001) also remained significant prognostic indicators, whereas sex was not statistically significant (P = 0.129) (Table 3).

### Targeted sequencing for FGFR2

**Fig 4:**
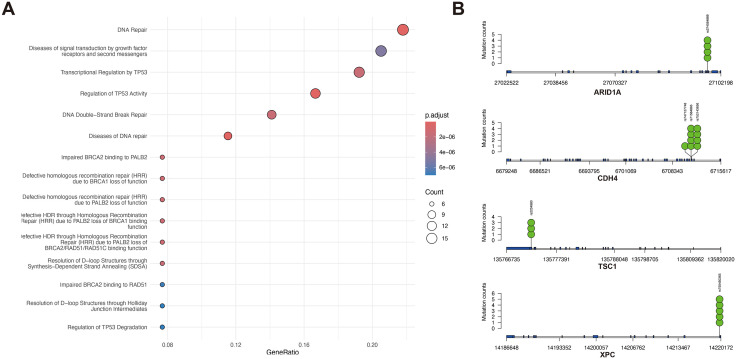
Results of targeted sequencing analysis in five patients with PCC-GC. **(A)** The top 15 enriched pathways based on the mutated genes. **(B)** Distribution of recurrent mutations across patients. ARID1A, CDH4 and TSC1 are genes related to genome instability, whereas the rs750450365 mutation in XPC was shared among all patients. The y-axis indicates the number of patients carrying the mutation. The x axis refers to the loci. Green dots represent a single mutation, whereas the blue thick lines refer to exons.

Although FGFR2 overexpression was common in PCCs in our cohort, targeted sequencing revealed no *FGFR2* amplification or pathogenic alterations. However, Case 1 had a pathogenic TP53 mutation, whereas Case 2 had a pathogenic TP53 mutation along with copy number alterations involving multiple chromosomes and amplifications of FGF3, FGF19, and CCND1. These alterations have been reported in intestinal-type adenocarcinomas [10]. Case 3 had pathogenic mutations of *MSH6* and many frameshift mutations, thus classified as an MSI-High adenocarcinoma. Only case 5 harbored a pathogenic mutation in *CDH1*, which is a conventional pathogenic alteration of PCC-GC (Table 4). A total of 82 genes that harboring at least one mutation in any patient were analyzed. They were subject to over-representation analysis (ORA) using ClusterProfiler (v4.12) [11] and R (4.4.1). A number of pathways were identified through enrichment analysis using the KEGG [12] and the Reactome [13] databases, respectively. The top 15 enriched Reactome pathways are shown in Fig 4A. The full list of the ORA results for Reactome, KEGG and GO:BP was provided in the S1 Data. The ORA analyses across Gene Ontology (GO:BP), KEGG, and Reactome databases suggested enrichment of pathways related to DNA damage response and repair dysfunction as the most significantly enriched biological process. Three genes related to genomic instability that shared the same mutations, and were annotated by ClinVar [14], are shown in Fig 4B (i.e., ARID1A, CHD4 and TSC1). The mutation rs750450365 in XPC (Xeroderma Pigmentosum, Complementation Group C) was shared by all five patients.

**Table 4:** Pathogenic alterations and copy number changes in selected FGFR2-overexpressing PCC cases.

Case	Gene	Nucleotide Alteration	Amino Acid Alteration	VAF (%)
1	*TP53*	c.637C > T	p.R213X	5.6
2	*TP53*	c.814G > C	p.V272L	15.8
3	*MSH6*	c.3261dup	p.F1088LfsX5	72.1
	*BRCA1*	c.1473_1497del	p.H493NfsX2	6.4
	*BRCA1*	c.1016del	p.K339RfsX2	9.9
	*BRCA2*	c.5073del	p.K1691NfsX15	5.2
	*ATM*	c.8395_8404del	p.F2799KfsX4	10.7
	*BLM*	c.1544dup	p.N515KfsX2	7.5
	*BLM*	c.1544del	p.N515MfsX16	8.9
	*CDK12*	c.2782del	p.E928NfsX18	14.3
	*FLCN*	c.1285dup	p.H429PfsX27	9.4
	*MEN1*	c.1561del	p.R521GfsX43	5.3
	*PMS2*	c.325del	p.E109KfsX3	7.9
	*RAD50*	c.2801del	p.N934IfsX6	5.7
	*RAD50*	c.2165dup	p.E723GfsX5	11.0
	*RAD50*	c.2165del	p.K722RfsX14	17.1
4	*BLM*	c.1544dup	p.N515KfsX2	8.4
	*HNF1A*	c.872dup	p.G292RfsX25	5.6
	*MSH3*	c.1148del	p.K383RfsX32	5.1
	*RAD50*	c.2801del	p.N934IfsX6	5.8
	*RAD50*	c.2165dup	p.E723GfsX5	6.6
5	*NSD1*	c.5581C > T	p.R1861X	16.4
	*CDH1*	c.1320 + 1G > A	–	28.3
Case	Gene	Copy number status	Copy number	Locus
1	16q	loss	1.43	16q
	19p	loss	1.57	19p
	19q	loss	1.59	19q
	21p	loss	1.31	21p
	FGF4	Del.	0.63	11q13.3
	FOXL2	Del.	0.71	3q22.3
2	CCND1	Amp.	7.27	11q13.3
	FGF19	Amp.	7.28	11q13.3
	FGF3	Amp.	6.67	11q13.3
3	15q	loss	1.64	15q
	19p	loss	1.46	19p
	19q	loss	1.56	19q
	4p	loss	1.55	4p
	FGF4	Del.	0.41	11q13.3
	FGF7	Amp.	6.42	15q21.2
	FOXL2	Del.	0.63	3q22.3
	HIST1H3B	Amp.	7.14	6p22.2
	PPP1R15A	Amp.	8.54	19q13.31
4	Not detected			
5	Not detected			

VAF, variant allele frequency; TP53, tumor protein p53; MSH6, MutS homolog 6; BRCA1, breast cancer 1, DNA repair associated; BRCA2, breast cancer 2, DNA repair associated; ATM, ATM serine/threonine kinase; BLM, BLM RecQ like helicase; CDK12, cyclin dependent kinase 12; FLCN, folliculin; MEN1, menin 1; PMS2, PMS1 homolog 2, mismatch repair system component; RAD50, RAD50 double strand break repair protein; HNF1A, HNF1 homeobox A; MSH3, MutS homolog 3; NSD1, nuclear receptor binding SET domain protein 1; CDH1, cadherin 1, E-cadherin; 16q, 19p, 19q, 21p, 11q13.3, 3q22.3, 15q, 4p, 15q21.2, 6p22.2, 19q13.31, chromosome number–arm–band location; FGF4, fibroblast growth factor 4; FOXL2, forkhead box L2; CCND1, cyclin D1; FGF19, fibroblast growth factor 19; FGF3, fibroblast growth factor 3; FGF7, fibroblast growth factor 7; HIST1H3B, histone cluster 1 H3 family member B; PPP1R15A, protein phosphatase 1 regulatory subunit 15A; c., coding DNA reference sequence; p., protein reference sequence; dup, duplication; del, deletion; fs, frameshift; X, stop codon; Amp., amplification; Del., deletion; loss, copy number loss.

## Discussion

This study confirms that FGFR2 positivity correlated with superior DFS and OS and was an independent prognostic factor for DFS. Moreover, FGFR2 overexpression was associated with early-stage disease and absence of lymph node metastasis.

Previous studies have demonstrated that altered FGFR2 signaling contributes to the pathogenesis of various solid tumors, including GC [15,16]. Several studies have explored the prognostic implications of FGFR2 expression [5,6,17–19]. In pancreatic cancer, Nomura et al. demonstrated that FGF10/FGFR2 signaling enhances cancer cell migration and invasiveness, contributing to poor prognosis [18]. Similarly, Sun et al. reported that high FGFR2 expression was observed in approximately 65% of patients with breast cancer and linked to reduced OS [19]. A meta-analysis by Kim et al. found that FGFR2 overexpression in GC was associated with deeper tumor invasion, lymph node metastasis, and advanced stage, suggesting a negative prognostic role [5]. While FGFR2 is associated with poor outcomes in several malignancies, including breast and pancreatic cancers, our findings suggest its prognostic role may vary by cancer type and biological setting [18–23]. The precise biological role of FGFR2 in malignancies remains unclear, with evidence indicating it may act as either a tumor promoter or suppressor depending on the cellular context, tumor microenvironment, and isoform expression [16,21–25].

Interestingly, while FGFR2 overexpression has previously been associated with tumor progression and therapy resistance in various malignancies [23], in our PCC‑GC cohort, FGFR2‑positive tumors were paradoxically associated with improved prognosis. Unlike previous studies, which evaluated FGFR2 expression across histologic subtypes, this study focuses exclusively on PCC-GC, potentially explaining the differing findings [5,6,8]. Previous molecular analyses already reported that PCC-GC is a highly heterogeneous disease that varies in both histologic presentation and genetic variation [24,25]. As a result, these conflicting results could be due to distinct characteristics of PCC-GC. Moreover, one possible explanation for this finding lies in the interaction between genomic instability and compensatory receptor tyrosine kinase signaling. In tumors harboring extensive DNA repair deficiencies—as suggested by the high burden of mutations in genes such as ARID1A, CHD4, and TSC1—cells may upregulate alternative survival pathways to maintain proliferative capacity. FGFR2 overexpression could represent such a compensatory or adaptive mechanism, wherein epithelial cells attempt to restore signaling balance in the context of pervasive chromosomal stress. However, given the very limited number of cases included in the targeted sequencing analysis, this hypothesis should be interpreted with caution and requires further validation. In this context, cells with partial DNA repair deficiencies may be more reliant on mitogenic signals from FGFR2, rendering them less aggressive in the absence of additional co‑drivers. Alternatively, FGFR2‑overexpressing tumors may arise in molecular subtypes with more intact DNA damage response mechanisms, distinguishing them from highly unstable, FGFR2‑negative tumors. Notably, NGS analysis showed that all five patients shared the rs750450365 mutation in the XPC gene. XPC is a key component of the nucleotide excision repair (NER) pathway, acting as a damage sensor, recognizing helix‑distorting DNA lesions caused by oxidative stress, UV light, or bulky adducts. Inactivation of XPC impairs global genome repair, allowing DNA damage to accumulate, especially in transcriptionally silent regions [26]. In the enrichment analysis, terms such as “DNA repair,” “DNA double-strand break repair,” and “Diseases of DNA repair” emerged with high significance (p.adj < 1 × 10 ⁻ ⁶). Pathways related to DNA repair were identified. However, given the limited sample size, these observations require cautious interpretation. Several mutated genes in our dataset are key regulators of chromatin state and genome maintenance. For instance, ARID1A, CHD4, and ASXL1 are members of chromatin remodeling complexes (e.g., SWI/SNF, NuRD), and their loss has been associated with impaired DNA repair and transcriptional deregulation in diffuse-type gastric cancer [27,28]. Similarly, mutations in TGFBR2 and TGFBR1, key transducers of TGF‑β signaling, have been implicated in defective cell cycle checkpoints and DNA damage tolerance. TSC1, CBL, and MAPK1 influence DNA repair indirectly through mTOR and MAPK signaling cascades, which intersect with genome surveillance pathways. Additionally, alterations in XPC, a critical component of nucleotide excision repair, and HLA‑A, involved in immune surveillance of DNA‑damaged cells, further support the idea that loss of DNA repair fidelity contributes to immune evasion and clonal evolution in PCC‑GC. The frequent mutation of ARID1A, in particular, is consistent with prior reports identifying it as a hallmark genomic lesion in PCC‑GC and other diffuse‑type gastric cancers [29]. Collectively, these findings may suggest a potential association between DNA repair-related alterations and PCC-GC, contributing to tumor heterogeneity, aggressiveness, and resistance to standard therapies; however, further studies in larger cohorts are required to confirm this observation. These observations may indicate a potential role of FGFR2 expression status in stratifying PCC‑GC subgroups by underlying genomic architecture, and further support the need to evaluate FGFR2 not only as a therapeutic target but also as a biomarker of molecular subtype and genomic stress adaptation.

Meanwhile, staging factor may influence the prognosis in PCC-GC. Indeed, while Hosoda et al. found no significant association between FGFR2 expression and survival outcomes in patients with stage II/III GC, a meta-analysis by Kim et al. reported that FGFR2 overexpression was significantly associated with advanced stage and reduced OS. These findings suggest that the prognostic relevance of FGFR2 expression may differ by disease stage [5,6,30], highlighting the complexity of FGFR2 signaling in cancer biology and the need for tumor-specific molecular marker evaluations rather than generalized interpretations across tumor types [6,22].

While FGFR2 overexpression was observed in approximately 10% of PCCs in our cohort, *FGFR2* amplification or pathogenic alterations were absent in the targeted sequencing analysis, although only a few cases were examined. Previous studies, including the FIGHT trial, have reported discrepancies between FGFR2 immunohistochemistry and sequencing results, suggesting that FGFR2 expression may be regulated by mechanisms beyond amplification or pathogenic mutations [7,23].

Additionally, PCC-GCs that underwent targeted sequencing demonstrated pathogenic alterations not commonly associated with conventional PCC-GCs (Table 4). This may partially explain the favorable prognosis of FGFR2-overexpressing PCC-GCs, given that they harbor intestinal-type adenocarcinoma-like genetic alterations and MSI-high tumors.

Recent developments of FGFR2-targeted therapies, such as bemarituzumab, has renewed interest in FGFR2 as a therapeutic target in GC. The FIGHT trial demonstrated promising efficacy of bemarituzumab in FGFR2-overexpressing tumors, suggesting that FGFR2 expression may predict response to targeted therapy [7,24,25]. Although our study did not evaluate treatment outcomes with FGFR2-targeted agents, identifying FGFR2 as a prognostic marker in PCC-GC may inform patient selection in future clinical trials involving FGFR2 inhibition in this subgroup.

This study has several strengths, including a large, homogeneous cohort of surgically resected PCC-GC cases, consistent histological classification, and comprehensive follow-up data. However, it also has limitations. As a retrospective, single-center analysis, the relatively small number of FGFR2-positive cases limits the statistical power to draw broad conclusions. Larger, multi-institutional studies with integrated molecular profiling are needed to clarify the biological significance and clinical utility of FGFR2 expression in this histologic subtype. Importantly, the targeted sequencing analysis was performed in a very small number of cases (n = 5), and therefore these findings should be considered exploratory and require validation in larger cohorts to ensure statistical reliability. In addition, *FGFR2* gene amplification or mutation status was not assessed alongside FGFR2 protein expression.

In conclusion, FGFR2 expression identified in a subset of PCC-GC was independently associated with improved DFS, suggesting its potential as a prognostic biomarker and therapeutic target in this histologic subtype. Further prospective studies and biomarker-driven clinical trials are needed to validate the role of FGFR2 in PCC-GC and assess the clinical efficacy of FGFR2-targeted therapies in this context.

## Supporting information

S1 Data Data‌‌. (XLSX)
